# CD4^+^/CD8^+^ mucosa-associated invariant T cells foster the development of endometriosis: a pilot study

**DOI:** 10.1186/s12958-019-0524-5

**Published:** 2019-10-15

**Authors:** Caihua Li, Zhimin Lu, Kaihuan Bi, Kangxia Wang, Yuping Xu, Peipei Guo, Ya Chen, Ping Zhou, Zhaolian Wei, Huanhuan Jiang, Yunxia Cao

**Affiliations:** 1 Reproductive Medicine Center, Department of Obstetrics and Gynecology, the First Affiliated Hospital of Anhui Medical University, Wanshui Road Nr.120, 230000 Hefei, People’s Republic of China; 2 Anhui Province Key Laboratory of Reproductive Health and Genetics, Hefei, People’s Republic of China

**Keywords:** Endometriosis, Mucosa-associated invariant T (MAIT) cells, IL-17, Peritoneal fluid

## Abstract

**Background:**

Immune dysregulation is one of the mechanisms to promote endometriosis (EMS). Various T cell subpopulations have been reported to play different roles in the development of EMS. The mucosa-associated invariant T cell (MAIT) is an important T cell subset in the pathogenesis of various autoimmune diseases. Evidence has indicated that there are three functionally distinct MAIT subsets: CD4+, CD8+ and CD4/CD8−/− (double negative, DN) MAIT cells. Till now, the associations between endometriosis and MAIT have not been studied. Our research investigates different MAIT subpopulations in peripheral blood (PB) and peritoneal fluid (PF) from EMS patients.

**Methods:**

Thirty-two EMS patients and eighteen controls were included. PB and PF were collected. Tests of cytokines in plasma and PF were performed by ELISA kit. Characterisations of MAIT were done by flow cytometry. MAIT cells have been defined as CD3 + CD161 + Vα7.2+ cells. Based on CD4 and CD8 expression, they were divided into CD8^+^MAIT, CD4^+^MAIT and DN MAIT.

**Results:**

Enrichments of MAIT cells, especially CD4 and CD8 MAIT subsets were found. Moreover, CD8 MAIT cells had a high activation in the EMS group. EMS patients produced higher level of IL-8/12/17 as compared to these from controls. On the contrary, control patients exhibited an impressive upregulation of DN MAIT cells, however, these DN MAIT cells from controls showed a higher expression of PD-1. Lastly, we performed the relevance analysis, and discovered that the accumulation of PB MAIT cells positively correlated with an elevated level of serum CA125 production in EMS group.

**Conclusion:**

These results suggest that different MAIT subsets play distinct roles in the progression of endometriosis.

## Introduction

Endometriosis (EMS) is a chronic disease which is characterised by the presence of endometrial cells outside the uterus [1, 2]. It affects up to 10% of women of reproductive age, who suffer symptoms, such as dysmenorrhea, chronic pelvic pain, pelvic inflammatory reactions as well as infertility [3, 4]. The pathogenesis of endometriosis has been studied for decades, however, a clear answer is still missing.

Many reports have indicated that the process of endometriosis is related to a dysregulation of the host immune and some researchers even consider EMS to be an autoimmune disorder [5–12]. On one hand, some studies have been focused on downregulation of anti-endometrial implants cells, such as NK cells, CD4^+^/CD8^+^ T cells, B cells and so on [5–8], on the other hand, some studies demonstrated that increased immunosuppressive cells could promote the progression of endometriosis, such as Tregs (regulatory T cells), TH2 (T helper) cells and even MDSCs (myeloid derived suppressor cells), which have been suggested recently to promote the implantation of endometrial tissue [13, 14]. All these evidences pointed to the fact that the impaired immune response exists in endometriosis.

Mucosal-associated invariant T (MAIT) cells are non-classical T lymphocytes characterised by a semi-invariant T cell receptor (TCR) which has been evolutionarily conserved [15–18]. This TCR consists of a restricted α chain (Vα7.2-Jα33 in humans and Vα19-Jα33 in mice) and one of the several β chains [16, 17]. It has been known that MAIT cells take part in both innate and adaptive immune response and can be found in peripheral blood and other tissues [18, 19]. However, till now there is no study about the relationship between MAIT cells and EMS, even though other kinds of T cells have been fully studied, such as TH17, TH1, TH2 and so on [10, 12, 20, 21]. In humans, MAIT cells have been defined as CD3^+^CD161^+^Vα7.2^+^ cells. Based on CD4 and CD8 expression, MAIT cells can be divided into CD8^+^MAIT cells and CD4/CD8^−/−^ (double negative, DN) MAIT cells which are the major population of MAIT cells, and CD4^+^MAIT cells which are only up to 2–11% of MAIT cells in human blood [18, 19]. MAIT cells have the ability to recognize microbial-derived vitamin B metabolites presented by the major histocompatibility complex class I (MHC I) - related protein 1 (MR1) and allow them to detect various strains of bacteria and yeasts in vitro and in vivo [22, 23]. Recently, multiple evidences showed that human MAIT cells with high expression of IL-18α could be activated by the pro-inflammatory cytokines IL-12 and IL-18 in a TCR independent manner [24–26]. Once activated, MAIT cells show cytotoxic properties, and secrete pro-inflammatory cytokines such as IL-17 and IFN-γ [23, 27]. It has been reported that both blood and peritoneal fluid from endometriosis patients are rich in IL12 and IL18 [28]. Therefore, we have the hypothesis that MAIT cells can be activated in endometriosis patients.

Next to the role of MAIT cells in mediating anti-microbial defenses, they have been implicated in the development of autoimmune and immunological diseases (multiple sclerosis, inflammatory bowel disease and inflammatory arthritis) [26, 29–31]. This suggests that MAIT cells may play a role in the manifestation of inflammatory responses in the absence of infection. Due to the functions of MAIT cells, many studies about different types of cancer suggested that MAIT cells have a great ability to destroy cancer cells [32–34]. However, some recent researches revealed that MAIT cells showed a potential to promote the progression of tumor by enhancing an immunosuppressive microenvironment which is induced by the cytokines produced by MAIT cells [35, 36]. For instance, it has been indicated that MAIT cells have the ability to facilitate the recruitment of MDSCs [37]. Since endometriosis has been reported to be a chronic inflammatory disease with malignant activities, what kind of role will MAIT cells play in endometriosis? Will they promote or prevent the development of endometriosis? Our study aims to assess the immune disorder of endometriosis by analyzing MAIT cell subpopulations and cytokine levels in the PF and PB from patients with endometriosis and controls. The present study reveals the relation between MAIT cells and endometriosis, thus finds out dysregulation of MAIT cells might be related to the immune disorder of endometriosis.

## Methods

### Patient selection

The study group comprised 32 patients with a diagnosis of endometriosis. They had their laparoscopy at the Department of Gynecology, the First Affiliated Hospital of Anhui Medical University from January 2018 to February 2019. Eighteen women with benign ovarian cyst (serous or dermoid) or uterine leiomyoma who underwent laparoscopy were recruited as control group. The selection of our control group is according to previous studies [9, 11, 38]. Table 1 displays the age, serum CA125 and menstrual days from both cases and control groups. The same medical team performed the surgeries for all patients. All patients had normal menstrual cycle, and the samples were taken when patients were at 5–14 days of their menstruation, and the antral follicular diameter from all patients were ≤ 10 mm which was measured via vaginal ultrasound before surgery. Peripheral blood (PB) was obtained shortly before the surgery. Peritoneal fluid (PF) was collected during laparoscopy. And peritoneal fluid samples with contamination of blood were discarded. Therefore, we got 29 PF samples from EMS patients and 10 samples from controls. The stage of endometriosis was scored according to the proposed revised American Society for Reproductive Medicine (rASRM) classification (Revised American Society for Reproductive Medicine classification of endometriosis, 1996) [39]. Table 1 shows the percentage of patients with different staged endometriosis: 7 - stage I (21.9%), 10 - stage II (31.2%), 8 - stage III (25.0%) and 7 - stage IV (21.9%) (Table 1).

**Table 1:** Characteristics of EMS and control patients

Baseline	All stage	EMS Early stage	Late stage	CG	*P* value
Subjects (*n*)	32	17	15	18	
EMS stage, *n* (%)					
I	7 (21.9%)	7			
II	10 (31.2%)	10			
III	8 (25.0%)		8		
IV	7 (21.9%)		7		
Uterine leiomyoma				10 (55.6%)	
Ovarian benign cysts				8 (44.4%)	
Age (years)^a^	32.6 ± 1.10	33.8 ± 1.53	31.3 ± 1.58	33.3 ± 1.23	NS
Menstrual days	27.6 ± 0.63	27.4 ± 0.88	27.9 ± 0.92	27.8 ± 0.76	NS
CA125^b^	83.0 ± 10.2	72.4 ± 14.9	95.1 ± 13.7	9.48 ± 0.86	< 0.0001

a) Age at the sample collecting; b) CA125 before surgery; *NS* Not statistically significant

### Sample preparation

Heparinised PB samples from all patients were taken in a sterile condition and centrifugated with the density gradient centrifugation by Biocoll (Biochrom, Berlin, Germany). The procedure was conducted according to the manufacturer’s instructions. Peripheral blood mononuclear cells (PBMCs) and plasma were collected. Clear PF samples were centrifuged with a speed of 2000 rpm for 10 min. PF supernatant and plasma was stored at − 80 °C. The PBMC and cell pellets from PF were counted using a Neubauer counting chamber and adjusted to 10^7^ cells. All cell samples were cryopreserved in the medium containing X-VIVO supplemented with 30% human serum and 10% DMSO at − 80 °C. All samples were stored until needed for analysis avoiding freeze-thaw cycles.

### Flow cytometry analysis

The staining procedure was the same as described before. In short, after thawing, samples were treated with FcR Blocking Reagent (Miltenyi Biotech, Germany) and stained accordingly with human anti-bodies (mAbs). They were anti-CD8-PerCP-Cy5.5, anti-CD3-APC-Cy7 (SP34–2), anti-CD4-FITC, anti-Vα 7.2-PE, anti-CD161-APC, anti-CD38-PE-Cy7, anti-CD279-PE-Cy7 and all of them were from BD Biosciences (Heidelberg, Germany). Then samples were washed. Acquisition was carried out by six-color flow cytometry using FACSVerse™ flow cytometry (BD Biosciences) with FACSuite software (BD Biosciences). Analyses of the data were made by FlowJo software (Tree Star, Ashland, OR, USA).

### Cytokines measurements in plasma and PF from patients by ELISA

IL-8, 12, 18, 17, MMP-9, INF-γ from the peritoneal fluid and plasma were analysed by ELISA kit (Multisciences Biotech, Hangzhou, China). The procedure was performed as the manufacturer indicated. Briefly, a polystyrene microplate of 96 well pre-coated with monoclonal antibody specific for each cytokine was used for each test. After final staining and washing, the optical density was determined.

### Statistical analysis

GraphPad Prism software (GraphPad Software, San Diego, USA) was used for statistical analysis. A one-way ANOVA test and an unpaired Student’s *t* test were performed respectively for multiple groups’ results or two groups’ results. Spearman analysis was performed for correlation test. Correlation coefficient is presented as *r*. A *p* value less than 0.05 was considered statistical difference.

## Results

### Presence of MAIT cells in PB and PF from patients

**Fig. 1:**
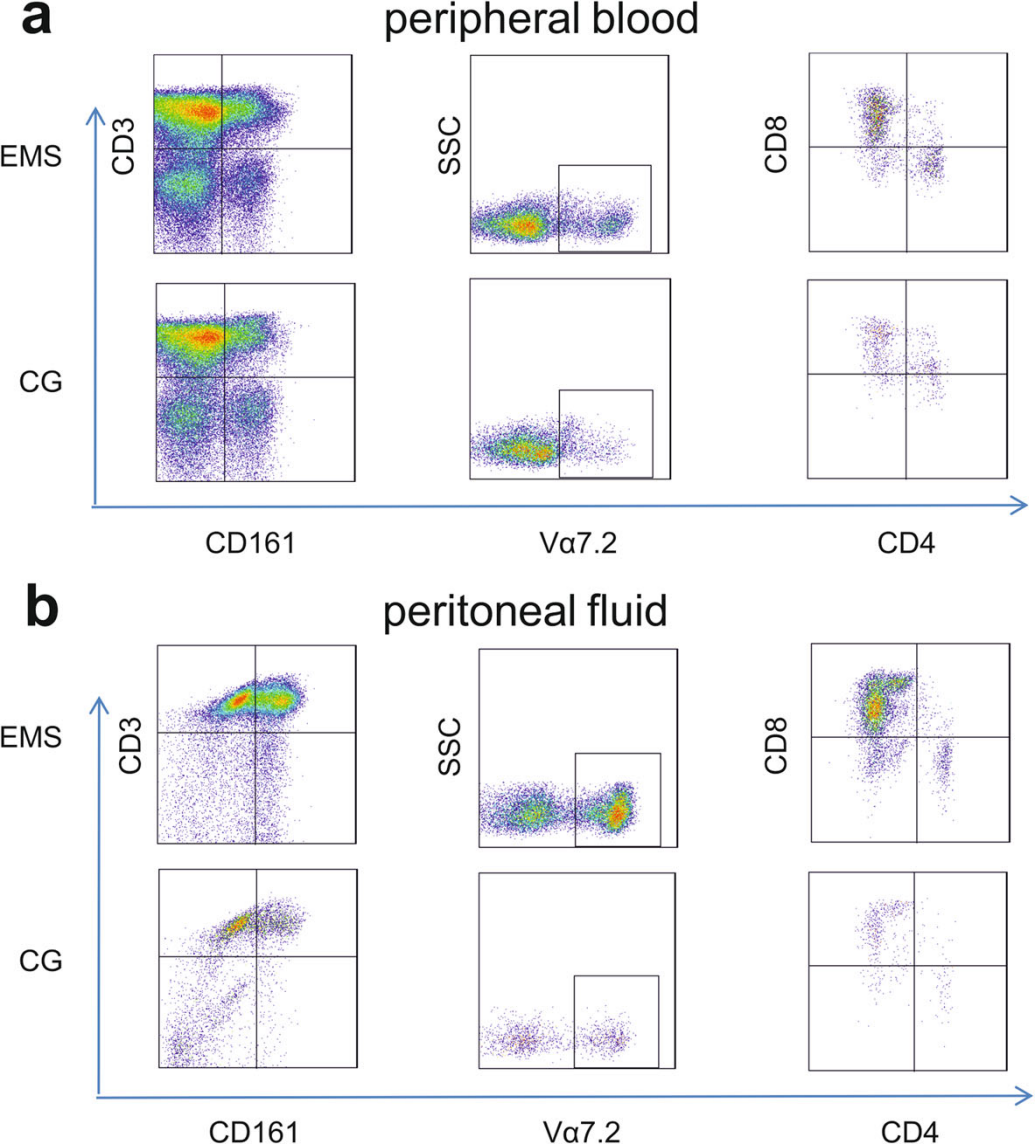
Cytometric characterisation of MAIT cells. Cells were from one EMS patient and one control. **a** and **b** exhibit PB sample and PF sample respectively. Live cells were gated (CD3^+^CD161^+^), and then Vα7.2^+^ cells were gated. The last gating step was based on CD4 and CD8, and three subsets were identified: CD8 MAIT cells, DN MAIT cells and CD4 MAIT cells. Data is shown as pseudocolor

To clarify the role of MAIT cells in the pathogenesis of endometriosis, we first tried to examine their existence in the PB (Fig. 1a) and PF (Fig. 1b) from endometriosis patients and controls. We characterised MAIT cells as CD3^+^CD161^+^Vα 7.2^+^ cells, and divided them into CD8^−^CD4^−^, CD8^+^CD4^−^ and CD8^−^CD4^+^ three subpopulations. Figure 1 shows the gating way of MAIT cells.

### Enrichment of the cytokines IL-8/12/17 in EMS patients

**Fig. 2:**
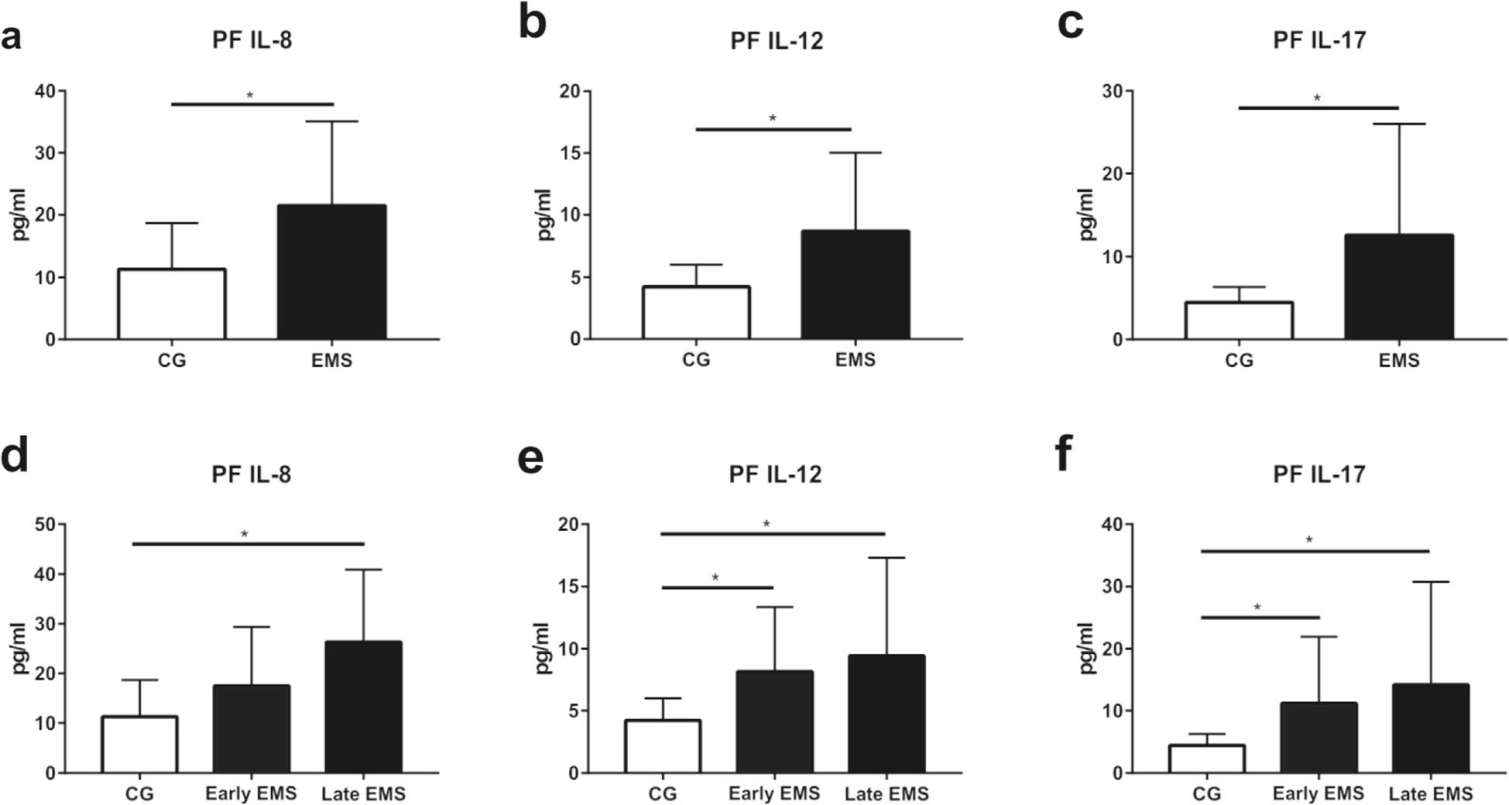
Comparation of cytokines in PF between EMS group and CG by ELISA. **a**–**f** Graphical representations display levels of IL-8/12/17 in PF from study and control groups. Concentrations are shown as the mean ± SEM. * indicates *P* < 0.05

To clarify if MAIT cells would be functional in the progression of EMS, we analysed IL-8, 12, 18, 17, MMP-9, INF-γ in plasma and PF samples from all patients using the ELISA kit. PF samples from EMS patients displayed a significant higher production of IL-8/12/17 (Fig. 2a, b, c) as compared to those from controls (11.30 ± 2.46 vs 21.50 ± 3.04, *P* = 0.0447; 4.23 ± 0.59 vs 8.70 ± 1.38, *P* = 0.0485; 4.50 ± 0.61 vs 12.56 ± 2.86, *P* = 0.0431) (Additional file 1: Table S1). When we divided all endometriosis patients into two groups: early staged group (stages I and II) and late staged group (stages III and IV), late staged EMS patients were found to produce increased levels of PF IL-8/12/17 (Fig. 2d, e, f) as compared to those from controls (11.30 ± 2.46 vs 26.37 ± 4.86, *P* = 0.0288), and the early staged EMS group showed higher secretion of PF IL-12 and IL-17 (Fig. 2e, f) (4.23 ± 0.59 vs 8.15 ± 1.50, *P* = 0.0220; 4.23 ± 0.59 vs 9.42 ± 2.62, *P* = 0.0356; 4.50 ± 0.61 vs 11.19 ± 3.10, *P* = 0.0406; 4.50 ± 0.61 vs 14.20 ± 5.24, *P* = 0.0499). We could not find any differences for cytokines in blood (Additional file 1: Table S1).

### Enhancement of different MAIT cells in EMS patients and controls

**Fig. 3:**
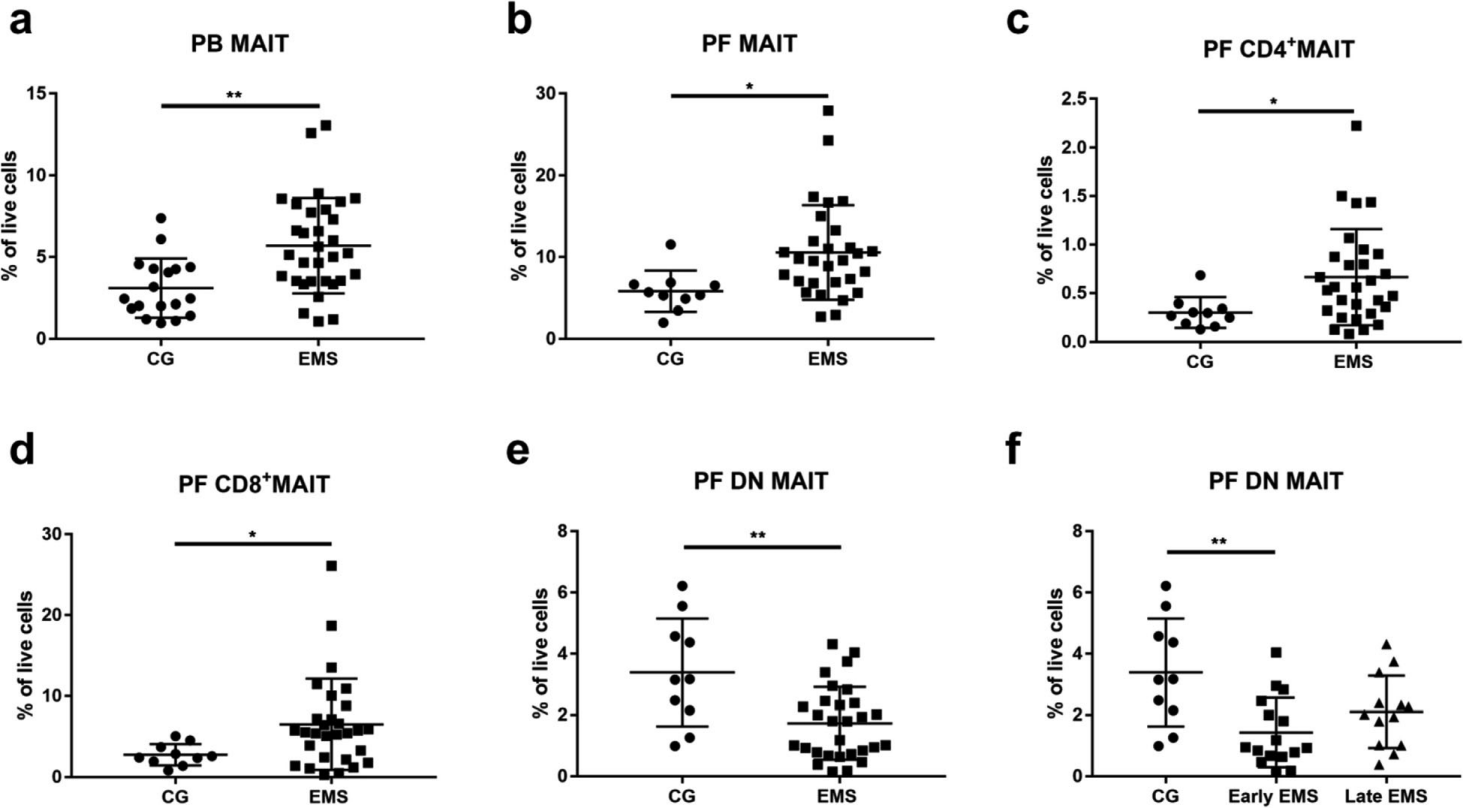
Different distribution of MAIT subsets in EMS and control patients. **a** and **b** show a significant increase of PB and PF MAIT cells (CD3 + CD161+ TCRV α 7.2+) from EMS group as compared with the control group. **c** and **d** display higher levels of PF CD4 and CD8 MAIT cells subpopulations from EMS patients as compared to those from controls. **e** and **f** exhibit a remarkable downregulation of PF DN MAIT cells in EMS group. Scatter plot represents the frequencies of MAIT cells in EMS group (*n* = 32 in PB and *n* = 29 in PF) and control group (*n* = 18 in PB and *n* = 10 in PF). * indicates *P* < 0.05 and ** indicates *P* < 0.01

To elucidate the involvement of MAIT cells in endometriosis development, we first analysed them in PB from all patients. Our results showed that EMS patients displayed a remarkably higher level of PB MAIT cells as compared to these from controls (Fig. 3a) (3.10 ± 0.43 vs 5.70 ± 0.52, *P* = 0.0013). Next we tried to find any difference for the three MAIT subpopulations: CD8^−^CD4^−^, CD8^+^CD4^−^ and CD8^−^CD4^+^MAIT cells. We could not find any difference for these three subpopulations in PB from different groups. Subsequently, we wanted to discover the MAIT cells’ patterns in the microenvironment of endometriosis. We characterised MAIT cells in the PF from all patients. The frequency of PF MAIT cells was detected to be increased in EMS patients as compared to controls (Fig. 3b) (5.82 ± 0.79 vs 10.56 ± 1.08, *P* = 0.0175). Out of the three subpopulations, CD4 and CD8 MAIT cells subsets rose significantly in EMS patients (Fig. 3c, d) (0.30 ± 0.05 vs 0.67 ± 0.09, *P* = 0.0295; 2.77 ± 0.42 vs 6.52 ± 1.05, *P* = 0.0454), Contrary to these results, EMS patients, especially the early stage group showed a dramatic deletion of DN MAIT cells (Fig. 3e, f) (3.39 ± 0.56 vs 1.73 ± 0.22, *P* = 0.0018; 3.39 ± 0.56 vs 1.43 ± 0.28, *P* = 0.0023). We also found that there were significant differences for the proportion of these three MAIT subpopulations in PB and PF from patients with EMS and controls (Table 2).

**Table 2:** Frequencies of MAIT subpopulations in PB and PF

		CD8 MAIT	DN MAIT	CD4 MAIT	*P* value
PB (%)	CG	2.45 ± 0.34	1.42 ± 0.19	1.03 ± 0.09	0.0002^a, b^
	EMS	2.50 ± 0.33	1.70 ± 0.24	1.29 ± 0.13	0.003^b^
PF (%)	CG	2.77 ± 0.42	3.39 ± 0.56	0.30 ± 0.05	< 0.0001^b, c^
	EMS	6.52 ± 1.05	1.73 ± 0.22	0.67 ± 0.09	< 0.0001^a, b^

Percentages presented as mean ± SEM

^a^Group A significantly different from Group B,

^b^Group A significantly different from Group C,

^c^Group B significantly different from Group C

The comparison between endometriosis patients and controls are displayed in Fig. 3

### Functional activation of MAIT cells from EMS patients and rapid consumption of MAIT cells in controls

**Fig. 4:**
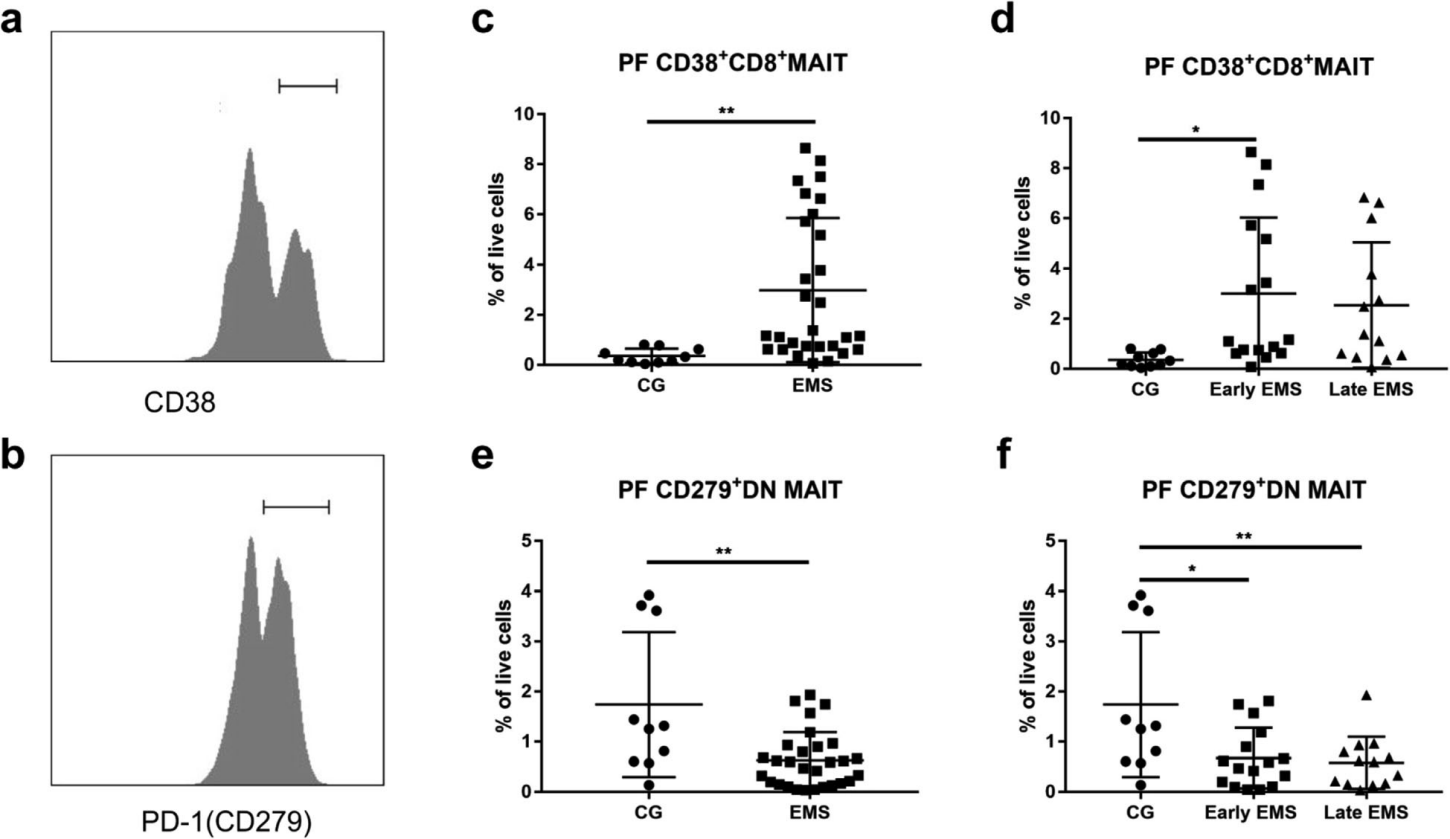
Activation and exhaustion of MAIT cells in peritoneal microenvironment from different groups. CD38 and PD-1 were used as a marker for activation of MAIT cells and exhaustion of MAIT cells respectively (**a** and **b**). All EMS patients and early stage group have higher expression of CD38 on CD8 MAIT subset (**c** and **d**). The control group shows a significantly higher level of PD-1 expression on DN MAIT cells as compared to those from EMS patients (**e** and **f**). * indicates *P* < 0.05 and ** indicates *P* < 0.01

At the end, we tried to find the activities of MAIT cells. CD38 was used to be a marker of MAIT cells activation and PD-1 was used as a marker of MAIT cells dysfunction (Fig. 4a, b) [22]. The expression of CD38 was elevated in the CD8^+^ MAIT cells in PF from EMS group (Fig. 4c) (0.36 ± 0.09 vs 2.98 ± 0.53, *P* = 0.0071), especially the early stage group as compared to these from controls (Fig. 4d) (0.36 ± 0.09 vs 3.00 ± 0.76, *P* = 0.0282), whereas, the control group showed DN MAIT cells with an increased expression of PD-1 in PF as compared to those from EMS patients (Fig. 4e) (1.74 ± 0.46 vs 0.63 ± 0.10, *P* = 0.0012), both early and late stage groups (Fig. 4f) (1.74 ± 0.46 vs 0.67 ± 0.15, *P* = 0.0123; 1.74 ± 0.46 vs 0,58 ± 0.14,*P* = 0.0089).

### The correlation between different MAIT cells and related cytokines and CA125 in EMS patients

**Fig. 5:**
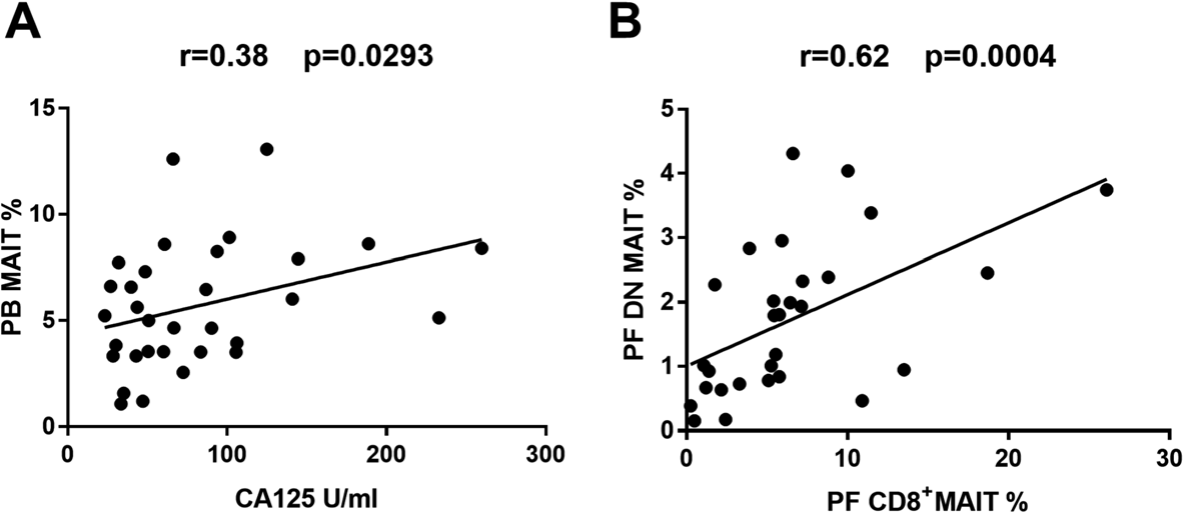
Correlation between PB MAIT cells and CA125, and association between DN MAIT cells and CD8 MAIT cells in PF from patients with endometriosis. **a** shows the correlation between PB MAIT cells and CA125 (*r* = 0.39 and *P* < 0.05). **b** exhibits the correlation between PF DN MAIT cells and PF CD8 MAIT cells (*r* = 0.62 and *P* < 0.01)

The serum CA125 level from EMS groups was remarkably elevated compared to the control group (Table 1). A positive association between the PB MAIT cells and CA125 from patients with endometriosis was discovered (*r* = 0.39 and *P* < 0.05, Fig. 5a), whilst DN MAIT cells were found to be positively related to CD8 MAIT cells in PF from endometriosis (*r* = 0.62 and *P* < 0.01, Fig. 5b). We could not find more correlation between other parameters.

## Discussion

Endometriosis has been extensively studied for decades, however the clear mechanisms for its pathogenesis are still poorly understood, due to the complexity of its initiation and process. One of the most known hypotheses is the retrograde menstruation theory of Sampson [40]. His theory indicated that endometrial cells (epithelium and/or glands) flowed through the fallopian tubes to the pelvis, and then implanted on other organs in the pelvis or even outside pelvis [40]. The haze is why only 10% of women have endometriosis, while retrograde menstrual flow happens to a high number of women [9, 10]. One explanation could be the microenvironment, especially the immunological and inflammatory factors.

An area of great interest in endometriosis is soaring in immunological activity and its function in development of this condition [6, 9, 10, 12]. The endometriosis-associated immunological reactions were well reported in previous studies indicating the abnormalities in the frequency and function of T cells and their associated cytokines [9, 10, 20]. Our study indicates that alterations in circulating and local MAIT cells may be one mechanism which causes immunological disorder in endometriosis.

MAIT cells are abundant in the host immune system [17, 41]. As the name implies, they reside in the mucosa, but they are also found in other organs or tissues such as the peripheral blood, lymphoid tissues, lung and liver [23, 30]. We are the first study to analyse them in the peritoneal fluid. Firstly, we found that MAIT cells also exist in the peritoneal fluid. They are CD8^+^CD4^−^MAIT cells, CD8^−^CD4^−^MAIT cells, and CD8^−^CD4^+^MAIT cells. In accordance with other studies, our results showed that for endometriosis patients in both PB and PF the CD8 MAIT cells were the major subpopulation and CD4 MAIT cells were the minority, with DN MAIT cells in between (Fig. 1 and Table 2). As IL-17 producing cells, MAIT cells have been reported to have a similar function of TH17 cells [23, 30]. Multiple studies have discovered that TH17 cells are enriched in EMS patients and play a critical role in the progression of EMS [10, 20, 42]. Therefore, we assumed that augment of MAIT cells could also contribute to the pathogenesis of EMS, and our results showed that EMS patients had increased frequencies of MAIT cells in PB and PF. Furthermore, we found that PF IL-17 was higher in EMS patients as compared to controls, which was identical to other reports [20].

MAIT cells and their ability of IL-17 production were previously studied in many autoimmune diseases and immunological disorder, such as multiple sclerosis, inflammatory arthritis, Type 1 diabetes, primary Sjogren’s syndrome and so on [26, 27, 30]. It was well studied that IL-17 was increased and took part in the pathological process in the above-mentioned diseases [30, 31]. Meanwhile, some researches also suggested that MAIT cells were equipped to launch TH17-skewed immune reactions [30]. Taken together, MAIT, TH17 and IL-17 could initiate a pro-inflammatory condition and even induce an immunosuppressive microenvironment. Interestingly, Rudak and his colleagues proposed that MAIT cells may promote the tumor development in tumorigenesis due to their potency of inducing MDSCs [37]. However, the relation between these cells needs further investigation. Moreover, our findings revealed that PF samples from EMS patients had elevated levels of IL-8 and IL-12 (Fig. 2). These results give further evidences that MAIT cells can be activated and function in endometriosis. Unfortunately, we could not find any association between measured cytokines and the frequencies of MAIT cells. Although, we discovered that in peripheral blood there was a positive relation between frequencies of MAIT cells and the level of serum CA125. Another study also found that TH17 cells positively correlated with serum CA125 [20]. This result might give a further hint that increased levels of MAIT cells are associated with the severity of endometriosis.

As mentioned before, there are three subpopulations of MAIT cells, and they were reported to have different functions and participate in various diseases [43–45]. The present study showed an interesting outcome. In our study, we found increased PF CD4 and CD8 MAIT cells in EMS group and highly activated CD8 MAIT cells in EMS group and early EMS group. In contrast, there was an elevation of DN MAIT cells within control group as compared to EMS patients, both early and late stage groups. One recent study by Dias et al. indicated that DN MAIT cells might derive from CD8 MAIT subset, but with different functions. In their study, they pointed out that DN MAIT cells had less cytolytic effect and were more prone to apoptosis [44]. Similar to their study, there was a positive relation between CD8 MAIT cells and DN MAIT cells. We also found that DN MAIT cells had a high expression of PD-1 in control group. Meanwhile, these two subsets of MAIT cells play opposite roles in endometriosis. One explanation would be that DN MAIT cells function as guardians in the immune system, and unlike the two other subsets, they exhaust quickly without producing overloaded pro-inflammatory cytokines. However, further researches about these mechanisms are needed.

## Conclusion

Our study revealed the role of MAIT cells in endometriosis and different profiles of their three subpopulations (i.e. CD8 MAIT, CD4 MAIT and DN MAIT cells). The outcomes of our research have identified that the disorder of MAIT cells might contribute to the immune dysregulation of endometriosis patients. CD4 and CD8 MAIT cells could be drivers in the development of in endometriosis, whereas DN MAIT cells might be protectors for the host. Therefore, manipulation of these cells might open new therapeutic strategies in the future.

## Supplementary information

**Additional file 1: Table S1.** Cytokine levels in PB and PF.

## Data Availability

The data supporting the conclusions of this article are available from the corresponding author on reasonable request.

## Abbreviations

CG: Control group
DN: Double negative
EMS: Endometriosis
IFN: Interferon
IL: Interleukin
mAbs: Human anti-bodies
MAIT cells: Mucosa-associated invariant T cells
MDSCs: Myeloid derived suppressor cells
MHC: Major histocompatibility complex class
MMP: Matrix metalloprotein
MR: MHC I-related protein
NK: Natural killer cell
PB: Peripheral blood
PBMCs: Peripheral blood mononuclear cells
PD: Programmed cell death protein
PF: Peritoneal fluid
rASRM: revised American Society for Reproductive Medicine
TCR: T cell receptor
TH: T helper
Tregs: Regulatory T cells
