# Supplementary material for: CD4+/CD8+ mucosa-associated invariant T cells foster the development of endometriosis: a pilot study
Source: Reprod Biol Endocrinol. 2019 Oct 15;17:78. doi: 10.1186/s12958-019-0524-5 (PMC6794756; doi:10.1186/s12958-019-0524-5)
Supplement: Supplementary file 1 — Additional file 1: Table S1. Cytokine levels in PB and PF. [file 12958_2019_524_MOESM1_ESM.docx]

Supplementary Table 1 - Cytokine levels in PB and PF.

|  |  | IL-8 | IL-12 | IL-17 |
| --- | --- | --- | --- | --- |
| PB | CG | 27.85 ± 6.95 | 4.99 ± 0.87 | 8.58 ± 4.85 |
|  | EMS | 19.01 ± 6.00 | 4.89 ± 1.75 | 3.84 ± 0.89 |
| PF | CG | 11.30 ± 2.46 | 4.23 ± 0.59 | 4.50 ± 0.61 |
|  | EMS | 21.50 ± 3.04 | 8.70 ± 1.38 | 12.56 ± 2.86 |
| Unit is pg/ml. | | | | |
